# SHARE-ENV: A Data Set to Advance Our Knowledge of the Environment–Wellbeing Relationship

**DOI:** 10.1021/envhealth.3c00065

**Published:** 2024-01-05

**Authors:** Catarina Midões, Enrica De Cian, Giacomo Pasini, Sara Pesenti, Malcolm N. Mistry

**Affiliations:** † Department of Economics, Ca’ Foscari University of Venice, Cannaregio 873, Fondamenta S.Giobbe, 30121 Venezia ,Italy; ‡ Institute of Environmental Science and Technology of Universitat Autònoma de Barcelona, Carrer de les Columnes s/n, 08193 Cerdanyola del Vallès, Barcelona, Spain; § Fondazione CMCC, RFF-CMCC EIEE, Via Della Libertà 12, 30175, Marghera, Venice, Italy; ∥ European Central Bank, Sonnemannstrasse 20, 60314 Frankfurt am Main, Germany; ⊥ Environment and Health Modelling (EHM) Lab, Department of Public Health, Environments and Society, London School of Hygiene and Tropical Medicine (LSHTM), Keppel Street, London WC1E 7HT, United Kingdom

**Keywords:** climate change risk, environmental impacts, climate adaptation, population health, longitudinal data

## Abstract

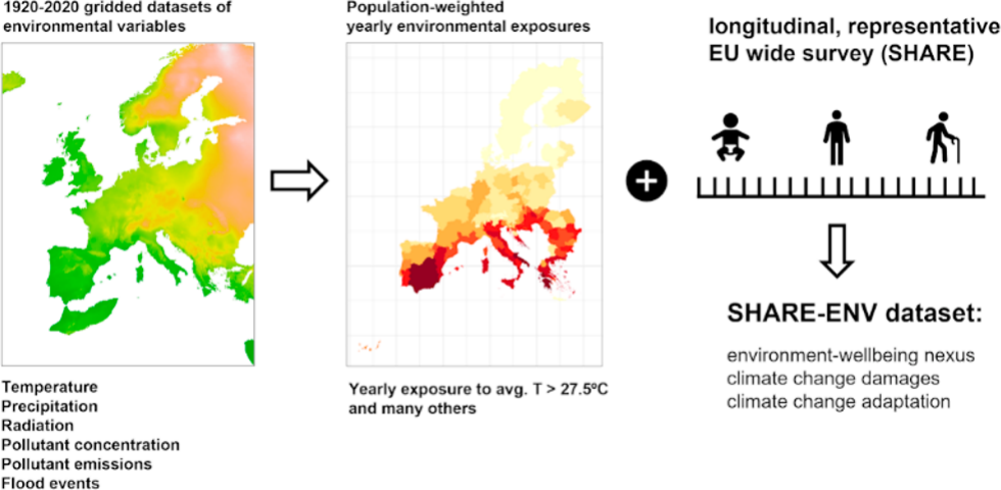

Climate change interacts with other environmental stressors and vulnerability factors. Some places and, owing to socioeconomic conditions, some people, are far more at risk. The data behind current assessments of the environment–wellbeing nexus is coarse and regionally aggregated, when considering multiple regions/groups; or, when granular, comes from ad hoc samples with few variables. To assess the impacts of climate change, we require data that are granular and comprehensive, both in the variables and population studied. We build a publicly accessible data set, the SHARE-ENV data set, which fulfills these criteria. We expand on EU representative, individual-level, longitudinal data (the SHARE survey), with environmental exposure information about temperature, radiation, precipitation, pollution, and flood events. We illustrate through four simplified multilevel linear regressions, cross-sectional and longitudinal, how full-fledged studies can use SHARE-ENV to contribute to the literature. Such studies would help assess climate impacts and estimate the effectiveness and fairness of several climate adaptation policies. Other surveys can be expanded with environmental information to unlock different research avenues.

## 1 Introduction

The Glasgow Climate Pact adopted at the 26th United Nations Conference of Parties (COP26) calls for an improved understanding of the geography of climate change impacts, related adaptation needs, and response options. Climate and environmental risks affect people in different ways, depending on the context in which they live and on their individual characteristics.^1,2^

Analyses conducted at the territorial level provide important insights into the regional dimensions of climate and environmental impacts, but even subnational studies do not address how environmental risk affects the wellbeing of different groups within wider geographies over time and across generations.^3^ Moreover, they cannot create the quasi-experimental settings needed to evaluate the effectiveness of adaptive behaviors. A recent review on the climate adaptation literature^4^ underscores the lack of quantitative assessments of climate adaptation. Out of 1,628 papers on climate adaptation reviewed, only 30 articles present primary quantitative evidence on the effectiveness of adaptation, and only 15 articles provide quantitative estimates. At least three reasons can explain the paucity of studies in climate adaptation evaluation: the lead time between actions and effects; the difficulty in causally linking exposure with the outcome; and the difficulty in measuring outcome variables. The existing evidence in the empirical economic literature is still piecemeal and confined mostly to the United States; and, moreover, to a few outcome and adaptation variables, namely, mortality and air-conditioning,^5^ and learning and air-conditioning.^6^ In the epidemiology field, a few studies provide conflicting evidence on the ability of air-conditioning to reduce mortality.^7,8^

Wellbeing is a complex and contested concept.^9^ Health-related dimensions that incorporate physical health and mental health, perceived and objectively measured, are unambiguously some of its defining dimensions. Vulnerability links to numerous individual characteristics, among them age, gender, education, and socioeconomic status, and to many health-related dimensions, such as pre-existing health conditions, lifestyles, and awareness of risk. Individuals can act to reduce the impacts of climate change only if they have access to safe housing, access to appropriate healthcare, and the ability to devote resources to unforeseen expenses in times of need.

We argue that granular, individual-level, representative longitudinal survey data can be expanded with variables on environmental hazards, to advance the causal assessment of both environmental impacts and adaptation interventions. This strategy can provide the much-needed information for evaluation of climate actions and the pursuit of climate justice.^10^ Longitudinal studies, following individuals over long periods, can uncover causal relationships among exposure, vulnerability, and policy interventions and actions. Built to represent populations of interest and provide a wide wealth of data, these studies also hold more promise than current causal inference studies, which resort to ad-hoc samples.

We show the potential of this strategy by expanding on the longitudinal Survey on Health, aging, and Retirement in Europe (SHARE), a European Union (EU)-funded initiative. The SHARE survey interviews approximately 120,000 individuals every two years since 2004 and is representative of 50+ EU-27 residents (plus Israel). Importantly, two specific interviews, conducted in the third and seventh waves (2008, 2016), called SHARELIFE, reconstruct retrospective life history, providing year on year information on respondents’ life conditions, health history, healthcare use, and working lives. We expand on SHARE by building variables on individual-specific yearly and cumulative exposures to different environmental hazards. The result is the SHARE-ENV data set (currently available in an online repository). We demonstrate that these data can be used to study relationships between environment and wellbeing and, ultimately, to advance the climate adaptation and climate policy literatures. The data can uncover links between climate change and human health that are usually hidden in purely regional analyses.

We use the SHARE-ENV data set and develop several illustrative analyses as proof of concept of its potential to shed light on the heterogeneity and ramifications of climate change impacts. The remainder of the article is organized as follows. Section 2 describes the data sources and the construction of SHARE-ENV. Section 3 provides examples of the type of relationships that can be explored with SHARE-ENV through cross-sectional and longitudinal multilevel regressions. We consider impacts on labor productivity, whose reduction is a well-established climate change impact, and on health and well-being, on which SHARE provides extensive information. In section 4, we discuss in more detail the advantages of SHARE-ENV which become visible through our illustrating examples. We describe why and how full-fledged analyses based on SHARE-ENV could give substantive contributions to the literature. In section 5, we discuss the potential of SHARE-ENV for future research, focusing on its potential to study adaptation.

## 2 Methods: SHARE-ENV Data Set

Our database combines a set of environmental hazards, extreme temperatures, solar radiation exposure, heavy precipitation, average and/or high concentration of ozone, nitrogen dioxide, and two particulate matter measurements PM_2.5_ and PM_10_ and flood events, with a comprehensive set of variables on individual-level health, on behavioral risks, and on risk-averting behaviors at different points in life in Europe, from the SHARE database.

SHARE is a longitudinal stratified sample representative of 50+ EU-27 residents (plus Israel). It contains approximately 120,000 individuals and 300,000 interviews.^11^ The regular panel waves (2004–2019) of SHARE follow individuals (and their spouses) over time. Respondents are interviewed every two years. In addition, the SHARELIFE modules (waves 3 and 7 in 2008 and 2016) reconstruct the retrospective life history of respondents. These histories include key focal points, such as the age at which a person left school, the dates when the person started and ended any given job, the dates of the onset of any illness, and details about changes in housing circumstances and family composition. Importantly, the retrospective accommodation models provide information on all regions where individuals have lived throughout their lives, which we explore to build exposure variables.

### 2.1 Main Outcomes of Interest

The SHARE database contains numerous variables which can be used to characterize the impacts of climate change on an array of morbidity types, subjective health indicators, and clinical and subclinical health outcomes. SHARE quantifies perceived health status at the individual level from poor to excellent. Clinical objective health indicators can be retrieved through questions on whether an individual has ever been diagnosed or bothered by a disease, whether he or she is taking drugs for certain illnesses, and the age of the onset for a range of illnesses, such as heart attack, stroke, high blood pressure, asthma, lung disease, cancer, diabetes, arthritis, Alzheimer, Parkinson, mental disorders/depression among others.

Respondents provide information on up to three periods of prolonged ill health throughout life, with a start and an end year, and what health conditions were responsible for such periods. Questions about the severity of illness include whether they brought on negative consequences at work, whether they limited social life and leisure activities, or whether they otherwise impacted the family negatively. From these SHARE primary data, we generate additional health variables to facilitate the analysis of environmental factors. We describe them in Table S3 in the Supporting Information.

There are also clinically measured health outcomes, some targeted to older age individuals. These include depression scores, cognitive scores for different cognitive functions, physical health measures (difficulties with Activities of Daily Living (ADL) and difficulties with Instrumental Activities of Daily Living (IADL), lung functioning, walking speed, grip strength, and dried blood spots).

Childhood health is considered separately. Beyond perceived childhood health status (variable takes values from 1 to 5, excellent to poor), other questions measure possible severity of health conditions during childhood. (Namely, if the respondents ever missed school for at least one month, if they were ever confined to their beds for at least one month, ever committed to a hospital for one month or longer, or ever hospitalized at least three times in a single year.) Respondents answer whether they had any of a list of illnesses, of note, infectious diseases, asthma, respiratory problems other than asthma, allergies, severe diarrhea, severe headaches, emotional problems, childhood diabetes, and heart trouble. Respondents provide information on illness onset and duration. (Unlike for health conditions during adult life, regarding childhood, respondents do not provide exact start and end dates for the illness, but state whether the condition lasted for at least one year, and whether it took place from 0 to 5 years old, from 6 to 10, or from 11 to 15 years old.)

In addition to morbidity and health outcomes, a wide range of other individual- and household-level characteristics are available. These include, for example, quality of housing, location of dwelling (big city, the suburbs or outskirts of a big city, a large town, a small town, a rural area or village), type of housing situation (e.g., owner versus renter), occupation including ISCO coding, education including ISCED codes, and job conditions. Information commonly collected in longitudinal surveys about income, wealth, material well-being, and migration is likewise available. Some variables of particular relevance for health outcomes are also available, namely, variables on behavioral risks (e.g., smoking, drinking; stress levels; parental behavioral risks). Several other research questions, outside the health/wellbeing framework, can be tackled using the wealth of information provided by SHARE, namely, those related to labor supply and labor productivity.

### 2.2 Construction of Environmental Variables

To generate variables on exposure to environmental hazards, we resort to high-resolution gridded data sets and the information derived from SHARE on where individuals have lived in each year of their lives, from birth until last survey participation. Individual location is provided in the retrospective accommodation modules of SHARELIFE and through the region in which the household was located at the moment of sampling in the regular waves. The regions are cantons in the case of Luxembourg and NUTS regions (Nomenclature of territorial units for statistics; the EU classification of the territory for regional statistics.) for the remaining EU countries, in their majority NUTS2 (see the Supporting Information for more details on the NUTS classifications used).

With gridded data sets of temperature, radiation, precipitation, pollutant concentrations and emissions, and flood events, we generate, first at the grid cell level, yearly variables on environmental hazards. From the high-resolution, daily, near-surface temperature, precipitation and radiation gridded-observational data E-OBS, made available by the European Climate Assessment & Data set (ECA&D) at 0.1° X0.1° resolution,^12^ we generate: bins of daily mean, minimum, and maximum temperature, average seasonal temperature, heating degree days and cooling degree days, yearly and seasonal average radiation and number of days with precipitation above 10 and 20 mm.

From the Dartmouth Flood Observatory (DFO) database^13^ we build variables on number of flood events and flood intensity. From the Copernicus Atmosphere Monitoring Service (CAMS) global reanalysis (EAC4) monthly averaged fields on pollutant concentration,^14^ we build average yearly concentration of PM_2.5_, PM_10_, and NO_2_, and yearly and summer average concentration of ozone. From the Emissions Database for Global Atmospheric Research (EDGAR, ver 5.0), made available by the European Commission Joint Research Centre (JRC),^15^ we build yearly emissions of PM_2.5_ and PM_10_. We elaborate on the choice and construction of variables in the Supporting Information.

**Figure 1:**
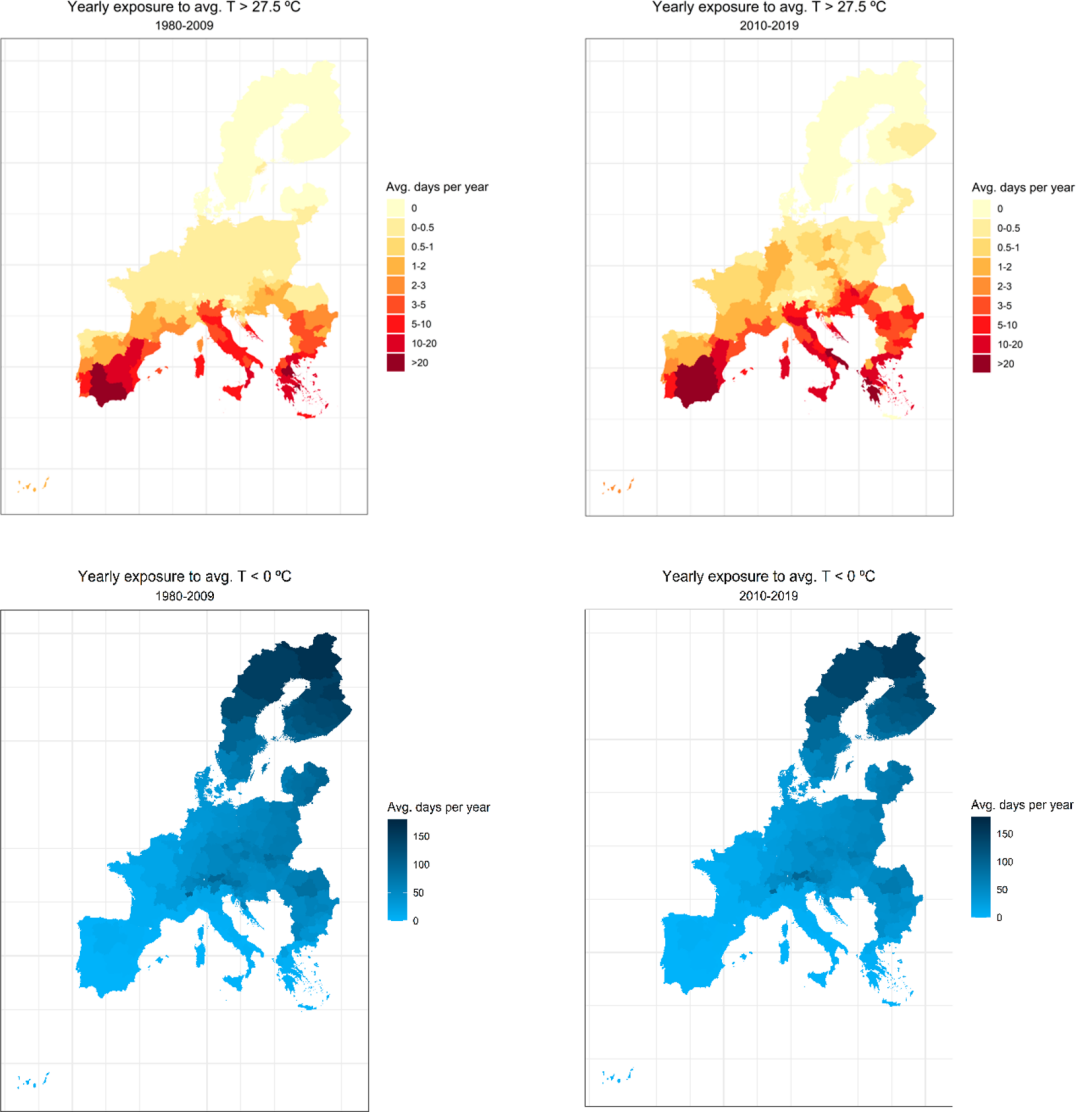
Selective environmental variables: Number of average annual days with daily average temperature above 27.5 °C (top) and with daily average temperature below 0° (bottom).

**Figure 2:**
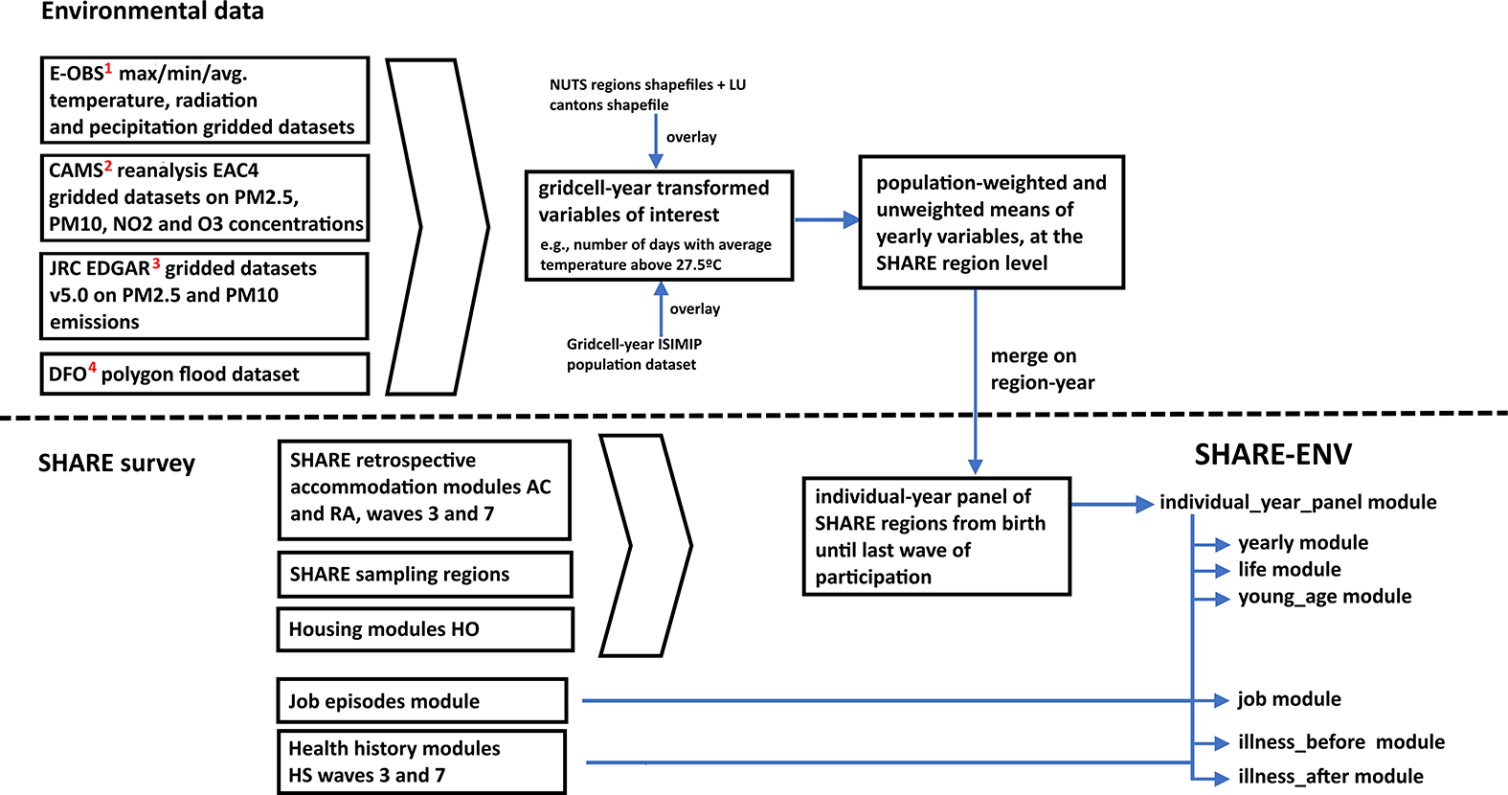
SHARE-ENV construction. Environmental data available at: (1) European Climate Assessment& Data set (ECA&D); (2) Copernicus Atmosphere Monitoring Service (CAMS); (3) JRC EDGAR v5.0 Global Air Pollutant Emissions; (4) Dartmouth Flood Observatory (upon request).

We aggregate these variables from grid cells to the regions reported by SHARE respondents using unweighted and population-weighted means. The next maps show average yearly bins of average temperature, specifically, the average number of days per year where average temperature was above 27.5 °C and below 0 °C for each SHARE region (Figure 1). We show one map for the average between 1980 and 2009 and one for the average between 2010 and 2019:

We merge these aggregate variables to SHARE respondents, based on yearly information on their residence, from birth until the last SHARE wave. From yearly variables, we construct cumulative variables, measuring exposure that had occurred from the time an individual was born until the wave in question and in critical periods, namely childhood. This process is summarized in Figure 2.

A second version of the data set, to be released after additional robustness checks, provides more granular geographical information. In such a version, we divide each NUTS region into five subregions and provide population-weighted average environmental exposure in big cities, suburbs, large towns, small towns, and rural areas of every NUTS region. This brings additional, within the region, variation.

### 2.3 Data Structure

The construction of the SHARE-ENV database is illustrated in Figure 2. The resulting SHARE-ENV database consists of seven modules, all of which are available in an online repository. The following table provides a short description of each of them (Table 1):

**Table 1:** SHARE-ENV Modules

module	description	unique ID	main purpose
individual_year_panel module	yearly exposure in years since birth up to the most recent participation in SHARE	individual, year	long-term effects
yearly module	yearly exposure in year of wave (and one and two years before the wave)	individual, wave	short-term effects
life module	rolling exposure throughout life	individual, wave	cumulative effects
young_age module	cumulative exposure over the first five, ten and 15 years of life	individual	effects of critical period exposure
job module	cumulative exposure during the years at one’s most recent job	individual	effects on labor supply and labor productivity
illness_before module	cumulative exposure during one-, three-, and five-year periods before the onset of illness	individual, illness-period	effects on disease onset
illness_during module	rolling exposure during periods of illness	individual, wave	effects on disease progression

Four of these modules, the individual_year_panel, the yearly module, the life module and the illness_during module, are longitudinal. The first module, individual_year_panel, refers to yearly variables (i.e., environmental-hazard exposure in a specific year, as opposed to cumulative exposure or averages over longer time periods). It is not merged with current-wave information and, instead, provides a full individual-year panel for the period from birth until most recent participation in SHARE. This data set can be of particular interest when merged with other retrospective modules of SHARE, such as the jobs-episode module. A long-term longitudinal analysis is then feasible.

The second module, the yearly module, has the same variables but merged with wave-on-wave information. For each individual-wave observation, we report environmental-hazard exposure in the year of that wave, in the year before, and in the year two years before, signaled by suffixes “t0″, “t_1bf,” and “t_2bf,” respectively. Such a module only provides information on the waves in which respondents participated (alongside the information from one year and two years immediately prior to those waves). This module is most suited for longitudinal analysis of short-term effects, exploiting wave on wave variation in exposure and outcomes. The life module is in all similar except it provides cumulative and average exposure variables instead of yearly variables to study cumulative effects of environmental factors.

The illness_before and illness_during modules include their own generated variables on illness length and intensity. These are best suited to study how environmental factors might trigger/accelerate disease onset and how they might affect disease progression. In the illness_during module, variables differ between waves only for individuals for whom the illness period intersects with the SHARE interview period. The young_age module is designed to study the impact of environmental factors during critical life periods. The job module is designed to study outcomes related to labor supply and labor productivity, which are known to be adversely affected by climate change.

## 3 Illustrative Analyses

We use the SHARE-ENV data set to illustrate relationships between environmental stressors and four types of subjective and objective outcomes. These examples use four of the seven different modules of SHARE-ENV: the life module, the young_age module, the job module, and the yearly module, respectively. The exact estimation equations are listed below as well as the definition of the variables used. Analyses (i), (ii), and (iii) are cross-sectional analyses, where we keep only one observation per individual, the last wave of participation in SHARE unless stated otherwise, while analysis (iv) on cognitive decline explores the panel component of the data set. We consider individual-level confounders. All cross-sectional analyses include country fixed effects. We estimate all regressions through Ordinary Least Squares (OLS) except for the analysis on cognitive decline, where we also resort to fixed effects estimation. To ensure estimates are robust to heteroskedasticity, we use White standard errors in the cross-sectional analyses and cluster at the individual level in the panel analysis.^16,17^

Health/wellbeing is one of the areas in which SHARE has a competitive advantage vis-à-vis other surveys. Three of our illustrative analyses use such outcomes, which are directly connected to environmental damages: (i) the prevalence of breathlessness; (ii) perceived health status through life, and (iv) cognitive decline. These examples are far from an encompassing analysis of possible research questions. Several other research questions can be tackled by using the wealth of information provided by SHARE. We provide a quick illustration, analysis (iii), where we consider the effect of temperature on an outcome connected to labor productivity: perceived comfort at one’s job. Results are summarized in Table 2 and presented in more detail in Tables S4–S6 in the Supporting Information.

**Table 2:** Association between Environmental Hazards, Health Outcomes, and Risk-Avoiding Behaviors^a^

	1. ever experienced breathlessness		2. young age (<15) perceived reported health		3. uncomfortable job		4. high cognitive decline	
	(0 = no, 100= yes)		(1 = poor; 5 = excellent)		(0 = no, 100 = yes)		(0 = no, 100= yes)	
exposure	avg PM_2.5_ conc. median (μg/m^3^)	0.19*** (0.001)	avg first 15 years exposure to negative temperature (# days)	+0.0002 (0.0004)	avg winter temperature	0.748*** (0.131)	difference in PM_2.5_ conc. median (μg/m^3^)	0.374*** (0.075*)*
	avg cum. lifetime exposure to negative temperature (# days)	4.42 × 10^–04^ (9.1 × 10^–03^)	avg first 15 years exposure to temperature >30 °C (# days)	0.003* (0.0015)	avg summer temperature	–0.268*** (0.100)	difference in heating degree days	–7.11e-04*** (5.31e-05)
	avg lifetime exposure to temperature >30 °C (# days)	–0.044* (0.024)	avg first 15 years solar radiation (W/m2)	0.002* (0.001)	avg radiation	0.015 (0.037)		
exposure × Individual characteristics					job is physical × average winter temperature	–1.18*** (0.156)		
					job is physical × average summer temperature	0.831*** (0.136)		
					job is physical × average radiation	0.135*** (0.026)		
individual confounders		Y		Y		Y		Y
country fixed effects		Y		Y		Y		Y

a Notes: Model 1 includes fixed effects of the International Standard Classification of Occupations (ISCO) (at the one-digit level). Model 3 includes fixed effects for the ISCO (at the one-digit level), the International Standard Classification of Education (ISCED), and the country. The corresponding questions to outcome variables 1 to 3 are the answers to the following questions or statement. Ever experienced breathlessness: “For the past six months at least, have you been bothered by any of the health conditions of breathlessness or difficult breathing?” Responses indicate whether they selected this symptom in any survey wave. Young age perceived health: “Would you say that your health during your childhood was in general excellent, very good, good, fair, or poor?” Responses were coded as follows: excellent 5, very good 4, good 3, fair 2, poor 1. ”My immediate work environment was uncomfortable (for example, because of noise, heat, crowding).” Answers were coded as follows: one ((has an uncomfortable job) for “Strongly Agree” or “Agree”; zero (does not have an uncomfortable job) for “Disagree” and “Strongly Disagree.” Model 4 outcome variable is whether the cognitive score of the list learning test decreased by more than 15% on a yearly basis between waves (other thresholds yield similar qualitatively results). It includes controls for the type of area of the house–whether a big city, the suburbs of such a city, a large town, a small town or a rural area. Exposure variables are unweighted, but weighted variables yield very similar results.

### 3.1 The Empirical Model

#### 3.1.1 Cross-Sectional Analysis

Our generic estimation equation for analyses (i), (ii) and (iii) is a multilevel cross-sectional linear regression between *y*_*i*_, an indicator of health/wellbeing outcomes observed for a given individual *i* in the wave of participation in the survey, and *K* average environmental variables ENV_*seq*_^*k*^, averaged over a sequence *seq* of regions where the individual has lived until the wave of participation:
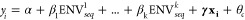
where *y*_*i*_ is measured with selected illustrative health/wellbeing outcomes:

1. Ever experienced breathlessness (100 if yes, 0 otherwise);
2. Perceived reported health (1 = poor, until 5 = excellent) at different points during the lifetime; 15 years of age, first wave of participation and last wave of participation;
3. Uncomfortable job (100 if yes, 0 otherwise).

and , where

ENV_*rt*_^*k*^ is the environmental variable *k* in year *t* for the smallest region *r* the individual reports living in in year *t*, from the beginning of the relevant period (*t* = *t*_0_) until wave of participation (*t* = *T*).

Our ENV_*seq*_^*k*^ variables are rolling averages, following individuals throughout the regions to which they move during their life. For these illustrative relationships, various indicators of environmental and climate risk have been chosen in relation to the specific outcome variable. We consider only one observation per individual. The period of interest determines the precise sequence *seq* considered for the rolling averages:

1. Episodes of breathlessness are related to average PM_2.5_ concentration, average number of days with temperatures above 30 °C and average number of days with temperatures below 0 °C. In this case, the sequence *seq* pertains to the period since birth until last wave of participation.
2. Perceived health status is related to the average number of days with temperatures above 30 °C, the average number of days with temperatures below 0 °C, and, in the case of childhood perceived health, average radiation. When we consider childhood health, the sequence *seq* pertains to the period since birth until 15 years of age. We consider two different periods for old age health, with *t*_0_ being birth and *T* either the first or the last wave of participation in SHARE.
3. Perception about whether one’s job is uncomfortable is related to average winter temperatures, average summer temperatures and average radiation. In this case, *t*_0_ is the year when the individual started the job and *T* is the last wave of participation while employed.

All specifications include a vector (*x*_*i*_) of individual level variables, which are possible confounders, specifically: age, household income and other measures of material deprivation, whether an individual had any illness at birth, Body Mass Index (BMI), whether an individual ever smoked, frequency with which the individual practices sports (1 = more than once a week, 2 = once a week, 3 = one to three times a month, 4 = hardly ever, or never) and whether the individual’s job is uncomfortable (1 = strongly disagree, 2 = disagree, 3 = agree, 4 = strongly agree). In the case of childhood health, we also include indicators of parental education, childhood abuse/neglect, and time spent living in urban areas. All specifications include country-specific fixed effects, *θ*_*c*_.

In analysis (iii), we demonstrate how to assess heterogeneity across groups by interacting certain variables with our environmental exposure variables. Specifically, we interact physical_*i*_, a binary indicator of whether the job of an individual is physically demanding, with the ENV_*seq*_^*k*^ variables (summer and winter temperatures and radiation). We resort to the following estimation equation:
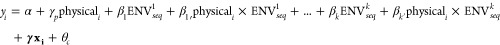


#### 3.1.2 Panel Analysis

For analysis (iv), we consider the relationship between the rate of cognitive decline and exposure to PM_2.5_. This analysis illustrates two different ways to use the panel nature of the yealy_module.

The first equation is estimated through pooled OLS, and includes lagged individual level variables, which we use to isolate factors commonly related to the rate of cognitive decline, such as general health, income, and education levels. The second equation represents an individual fixed effects model, which we estimate through the within estimator. We can only estimate the impact of time-varying variables, and include household income, age, exercise frequency, and a measure of depression.

We use two different estimation equations:
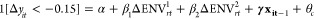
and
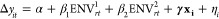
where:

- 1[Δ*y*_*it*_ < −0.15] is an indicator function taking value 100 if the annual decline in the cognitive score;
- *y*_*it*_ was higher than 15% and taking value 0 otherwise;
- *y*_*it*_ is the cognitive score of respondent *i*, from the words list learning cognitive test;
- η_*i*_ are individual fixed effects;
- ENV_*rt*_^1^ is the concentration of PM_2.5_ in region *r* in year *t*;
- ENV_*rt*_^2^ are heating degree days (HDD) in region *r* in year *t*.

### 3.2 Results

Having ever experienced breathlessness in one’s lifetime is positively related to average exposure to pollution (concentration of fine particulate matter, PM_2.5_), and the relative impact of actual exposure grows once one accounts for the relevant individual-level variables *x*_*i*_. A 10 μg/m^3^ higher daily average exposure to PM_2.5_ through life (an increase of approximately 2 standard deviations) is associated with a 1.9% point (p.p.) higher probability of experiencing breathlessness; for comparison, having ever smoked is associated with a 3.9% p.p. higher probability of breathlessness.

We find that perceived health in childhood is positively related to exposure to more frequent high temperatures. Such a relationship remains equally strong once we consider the significant positive effect of average solar radiation (positively correlated to high temperature extremes). If we consider an ordered probit model (as opposed to a linear regression), we find the same positive associations, as measured through average marginal effects (AME, not shown). Higher temperature and higher radiation increase the probability of reporting excellent health and decrease the probability of reporting poor, fair, or good health (not shown). A possible channel through which frequent high temperatures might have a positive impact on young age health is by allowing children to engage in more outdoor activities, a behavior we do not observe.

Cumulative exposure to extreme temperatures affects one’s *perceived health status* differently depending on when in one’s lifetime the question is posed. Exposure to both extremely high and extremely low temperatures is associated with worse perceived physical health in old age, unlike that in childhood. When we consider only the information provided in the first wave of individual interviews, only *extremely low* temperatures are significantly associated with worse health. By contrast, when we consider the most recent wave, in which individuals are considerably older (69 years old on average, 6 years older than the average age in their first wave), only *extremely high* temperatures are significantly associated with worse health status (see Supplementary Table S5). Ordered probit models, as opposed to linear regressions, confirm that these variables increase the probability of reporting poor and fair health and decrease the probability of reporting good, very good, or excellent health (in terms of AME, not shown).

We show that, for jobs that are physical, higher summer temperatures and higher summer radiation averages are associated with a higher probability of stating that one’s job is uncomfortable. For each additional degree in average summer temperature, individuals working physical jobs are 0.56 (0.831–0.268) p.p. more likely to report having an uncomfortable job. For this same type of job, in winter, milder/less cold temperatures are associated with a lower probability of having a job perceived as uncomfortable–each additional degree in winter temperature is associated with a −0.43 (0.748–1.18) p.p. change in the probability of feeling one’s job as uncomfortable. For nonphysical jobs, radiation does not have a significant effect, while a higher summer temperature reduces the probability of considering one’s job uncomfortable.

In our analysis regarding cognitive scores, in both specifications, we consider differences instead of levels of cognitive scores since a deterioration from one wave to another is expected; we are thus interested in differences in the rate of deterioration. We find that the higher the exposure is, the higher the cognitive decline. An increase of 10 μg/m^3^ in the average daily exposure to PM_2.5_ is associated with a 3.7 p.p. increase in the probability of showing large cognitive decline. We find a meaningful protective effect of several factors such as better general health and educational levels–for example, having primary school education instead of no schooling is associated with a 3.5 p.p. decrease in the probability of high cognitive decline. The same 10 μg/m^3^ increase in PM_2.5_, as estimated through the fixed effects model, is associated with an average decrease of 7 p.p. in cognitive scores (see Table S6 in the Supporting Information).

## 4 Discussion

The simplified analyses above show some of the characteristics of the SHARE-ENV data set which full-fledged analyses can explore to give meaningful contributions to the literature.

A first characteristic is that the outcomes of analyses (i), (ii), and (iv) on health and wellbeing are not the most commonly found in the literature. Regarding the association between health and pollutant concentration, a great part of the literature focuses on mortality.^18^ The same is true for the effects of extreme temperatures, focusing either on mortality or hospitalization rates.^19^ Using preclinical outcomes such as breathlessness has two main advantages. The most obvious is definitional: one can assess impacts that arise at an earlier stage. The second advantage, by comparison to healthcare data, is minimizing sample selection. Individuals who resort to healthcare are wealthier and sicker on average. Information on early stage cognitive decline is especially difficult to collect through healthcare data, as many individuals only resort to medical care in later stages of disease progression. We find statistically significant results (*p* < 0.05) in the three analyses conducted, showing associations between environmental hazards and nonacute negative health outcomes.

The literature on the relationship between pollution and cognitive decline is more limited than that on effects of pollution or temperature on morbidity or perceived health, though recent years have seen an increase in contributions. A recently published study^20^ contributes to the literature by considering multiple pollutants, multiple outcomes regarding cognitive capacity, and a large sample of individuals aged 45+ in metropolitan France, which “contrasts with most available studies which compare populations with relatively high exposure with those living in rural areas or small cities”. Through SHARE-ENV, a full-fledged analysis could likewise consider multiple pollutant and cognitive measures but with an even more extensive sample, spanning multiple EU countries and time periods.

In fact, the simplified analysis of high cognitive decline in the previous section already uses multiple time periods, i.e., the panel component of the longitudinal SHARE-ENV modules. We first exploit year-on-year variation on pollution concentration, finding a significant effect of pollution concentration on the likelihood of large cognitive decline. In that same analysis, we consider some possible risk factors for higher cognitive decline and find, as in the literature, that higher education levels and a higher level of physical activity are protective against cognitive deterioration. It is commonly assumed in the literature that year-on-year temperature variation is as good as random.^21^ Variation on pollution instead is only partly driven by as-good-as-random atmospheric conditions. While individuals are less likely to sort into regions based on yearly variation than on average values, we reduce this possible sorting bias by considering individual fixed effects. We find, once more, meaningful associations between variation in PM_2.5_ and faster cognitive decline while controlling for time variation in regional and individual factors.

Individual level analysis, even if cross-sectional, has great potential to advance the literature on the impacts of pollution and temperature on health outcomes whenever we can consider additional confounders. Important behavioral risk variables, such as whether an individual has ever smoked, are not easily found in regionally aggregated analysis nor in hospital admissions data sets, one of the most granular sources of data used in epidemiology literature. Socioeconomic variables, such as household income, are also not available at the individual level in such data sets and are often, at best, proxied by postal code indicators. Such data sets are thus still less granular and provide fewer variables than SHARE. Moreover, instead of being publicly available, they are usually licensed on a study-by-study basis due to their sensitive nature.

The importance of these confounders is clear in analysis (i), relating exposure to pollution and breathlessness: smoking behavior and household income are highly significant and correlated with regional level pollution. Once included, the impact of pollution becomes statistically significant. Additional variables, if they are important confounders, must be included to ensure unbiased estimation of effects. Even if they are not related to the environmental variables of interest, their inclusion can reduce unexplained variance and increase the power of the analysis.

Another advantage of using individual-level variables is the ability to put environmental hazards into perspective. As observed in analysis (ii), the magnitude of the association between higher temperatures/higher average solar radiation and improved childhood health is 2 orders of magnitude smaller than the association between childhood health and material deprivation (see Table S4 in the Supporting Information).

In analysis (ii), we looked at three different points—childhood: first wave of participation in the SHARE and last wave of participation. High temperatures are associated with better health in childhood and worse health only in the last wave of participation, when individuals are, on average, 69 years old. Such differences demonstrate the importance of considering different age groups separately for assessing vulnerability and ultimately design adaptation policies.

Other longitudinal surveys span a few decades of data collection, as well, but do not provide detailed retrospective life histories. A particularly unique feature of the SHARE-ENV data set is the ability to look at very early periods of life and at cumulative variables of exposure to hazards. Early life exposure is extremely relevant; for example, extreme temperatures are shown to have negative impacts on birth weight, which are then related to several negative health outcomes later in life.^22^ Disentangling the effects of short-term and long-term exposure to extreme temperature is also fundamental, as they have been shown to differ.^23^

Often studied climate change impacts other than reductions in well-being can also be revisited through SHARE-ENV, as the analysis on job comfort shows. Reductions of labor productivity are among the most widely discussed climate change impacts. The empirical literature on the topic is extensive yet, even when at the microlevel, is not without its issues. SHARE-ENV, given its detailed information about the sectors where individuals work, allows studying the heterogeneity of effects by sector. This differs from many microlevel analyses which are based on ad hoc samples (for instance, considering a sample of factories) and focus only on certain sectors, particularly agriculture or manufacturing.

The literature mostly considers aggregate measures of labor productivity, looks at one specific component of it, or, more rarely, considers jointly the number of hours worked and productivity during those hours together.^24^ With SHARE-ENV, it becomes possible to disaggregate specific mechanisms explaining why productivity is lower in the hours worked; comfort at the job is one example, but we can also consider attitudes toward work. It is also possible to look at channels driving the overall reduction in hours worked such as early retirement and illness onset.

In this quick example, we interacted exposure with whether a job is physical; finding such driver determines how temperatures and radiation affect comfort. Through SHARE-ENV, numerous similar heterogeneity analyses can be conducted to identify vulnerable groups.

## 5 Conclusions

The existing evidence in the empirical economic literature regarding adaptation is limited and focused on the United States. In the epidemiology field, a few studies provide conflicting evidence on the ability of air-conditioning to reduce mortality.^25,8^ As of now, only one study^6^ considers the mitigating effects of AC on learning outcomes in a quasi-experimental setting. SHARE-ENV, which provides information about AC ownership, can be used to study the mitigating effect of AC on varied health outcomes, encompassing dimensions of both mental and physical health. A forthcoming paper investigates this research question in detail.

Quasi-experimental evidence on heat alert systems, another adaptation policy, could also be expanded through SHARE-ENV. Reviewing the literature on the topic, we found only two papers which look at the effectiveness of heat warning systems in reducing morbidity (and 22 in reducing mortality) by considering hospitalizations.^26,27^ Comparatively, a study using SHARE-ENV could consider different outcomes or hospitalizations while adding more confounders on behavioral risk and economic conditions. Unlike for AC, the treatment variable must be constructed; that is, a variable on where and when heat alert systems were implemented and/or triggered must be built and merged with SHARE-ENV. A similar policy that requires additional quantitative evidence is the availability of climate refuges.

The quality of building insulation is thought to be an important and cost-effective strategy for climate adaptation. Yet, again, quantitative assessments are lacking. Variables on building stock can be merged to the SHARE-ENV data set, such as those provided in EUBUCCO.^28^ Other potential treatment variables relate to retrofitting policy interventions.

Other regional level adaptation measure whose effectiveness can be estimated through SHARE-ENV is the availability of green and blue spaces. Treatment variables must be built, yet they are easily attained through the same aggregation process we applied to our gridded data sets. Time-varying, gridded information on land use and cover is easily transformed into regional time-varying variables capturing the extension of public parks and public water bodies. The literature on the effects of green spaces on mental health generally (not as an adaptation channel specifically) is primarily qualitative. However, some quantitative studies exist. One to which a SHARE-ENV based analysis would resemble is Astell-Burt et al. (2014),^29^ who use the British Household Panel Survey (BHPS) and consider the relationship between general health and green space availability through longitudinal representative samples.

A great part of the adaptation literature focuses on econometric techniques to disentangle climate and weather effects and estimate adaptation by comparing the two.^30^ Yet, estimation is almost always conducted at the regional level. While some adaptation is place-based (citywide initiatives of climate refuges are an example), individuals greatly adapt to climate conditions. They do so physiologically and behaviorally. In SHARE-ENV, we know when individuals move to a new region—and what temperatures that region has been exposed to, as well as their cumulative life exposure to extreme temperatures. How many individuals who recently moved to new regions are affected by extreme temperatures compared to individuals who have always been there can help make inferences about the importance of behavioral and individual factors versus place-specific infrastructure and adaptation policies.

Merging environmental information with geographically localized, individual-level, longitudinal survey data can open new research avenues. We have demonstrated that this is the case for the SHARE-survey. The wealth of variables in SHARE and its representative, extensive EU samples allow researchers to disentangle heterogeneity of impacts of climate change and of effectiveness of adaptation policies. Moreover, it can help determine if policies favor specific socioeconomic groups, a crucial endeavor to design fair policies, both national and EU-wide. SHARE-ENV can help respond to the mission of climate justice by considering such factors. Better research on the connection between climate and health, which SHARE-ENV unlocks, is more important than ever as the COP28 Climate Change Conference moves to feature a Health Day for the first time since conception.

## Supporting Information Available

Details on environmental variables (climate data, pollution data, flood events data, regional aggregation and population weighting, cumulative variables); details on illness variables; extended regression results; summary statistics; supplementary references (PDF)
