# Supplementary material for: SHARE-ENV: A Data Set to Advance Our Knowledge of the Environment–Wellbeing Relationship
Source: Environ Health (Wash). 2024 Jan 5;2(2):95–104. doi: 10.1021/envhealth.3c00065 (PMC10877593; doi:10.1021/envhealth.3c00065)
Supplement: Supplementary file 1 — eh3c00065_si_001.pdf [file eh3c00065_si_001.pdf]

Supporting information

"SHARE-ENV: a dataset to advance our knowledge of the environment-wellbeing relationship"

Catarina Midões[a*,b], Enrica De Cian[a,c], Giacomo Pasini[a], Sara Pesenti[d], and Malcolm N. Mistry[a,e]

[a] *Ca' Foscari University of Venice, Department of Economics, Cannaregio 873, Fondamenta S.Giobbe 30121 Venezia, Italy*

[b] *Institute of Environmental Science and Technology of Universitat Autònoma de Barcelona, Carrer de les Columnes s/n, 08193 Cerdanyola del Vallès, Barcelona, Spain*

[c] *Fondazione CMCC, RFF-CMCC EIEE, Via Della Libertà 12, 30175, Marghera, Venice, Italy*

[d] *European Central Bank, Sonnemannstrasse 20, 60314, Frankfurt am Main, Germany*

[e] *Environment and Health Modelling (EHM) Lab, Department of Public Health, Environments and Society, London School of Hygiene and Tropical Medicine (LSHTM), Keppel Street* London *WC1E 7HT, United Kingdom*

*email: catarina.midoes@unive.it

Supporting Information

Appendix 1. Details on environmental variables S2

Climate data S2

Pollution data S3

Flood events data S4

Regional aggregation and population weighting S4

Cumulative variables S5

Appendix 2. Details on illness variables S14

Appendix 3. Extended regression results S15

Appendix 4. Summary statistics S21

Appendix 5. Supplementary references S23

List of Tables

Table S1 Environmental variables list S6
Table S2 Cumulative environmental variables list S10
Table S3 Generated morbidity variables in the SHARE-ENV dataset, modules illness_before and illness_during S14
Table S4 Extensive regression results of Main Text Table 1 .... S15
Table S5 Effect of temperature exposure on old age health at first wave of participation (younger age) and at last wave of participation (older age) S18
Table S6 Effect of $PM_{2.5}$ concentration on cognitive decline, fixed effects regression S20
Table S7 Summary statistics of variables in Table S4 .... S21
Table S8 Summary statistics of variables in Table S5 .... S22
Table S9 Summary statistics of variables in Table S6 .... S22

Appendix 1. Details on environmental variables

Climate data

The E-OBS gridded datasets[10] on temperature, radiation and precipitation, are the starting point for the climate data generated.

Temperature Bins

For yearly measures of the full temperature distribution, we focus on bins of temperature, i.e., the number of days in a year where the minimum (TN variable in E-OBS), mean (TG variable in E-OBS) and maximum (TX variable in E-OBS) temperature fall in one of the sixteen 2.5°C temperature intervals: <-5, -5 to -2.5, -2.5 to 0, 0 to 2.5, 2.5 to 5, 5 to 7.5, 7.5 to 10, 10 to 12.5, 12.5 to 15, 15 to 17.5, 17.5 to 20, 20 to 22.5, 22.5 to 25, 25 to 27.5, 27.5 to 30 and > 30, computed at the grid cell level. The use of temperature bins allows flexibility in considering the non-linear impacts of temperature on health and other variables of interest. We then assign the grid cells to the SHARE regions by employing a shapefile of the SHARE regions and geospatial routines from R packages sf and raster. We constructed a shapefile of the SHARE regions by resorting to EUROSTAT NUTS shapefiles (downloadable from EUROSTAT) and to a shapefile of Luxembourg cantons (downloadable from data.public.lu).

Once the bins are computed at grid cell level and georeferenced to a SHARE region, we aggregate them into two regional measures: median and mean. We also calculate the standard deviation between the cells of a SHARE region, given that, especially for larger regions, spatial variability might be substantial. Accordingly, the variable names end with '_median', '_mean' or '_std'.

Average (seasonal) temperature

We calculate the average annual temperature and the average seasonal temperatures – spring, summer, fall and winter - in the SHARE region where the respondent lived in a certain year. These are calculated for each grid cell as the average of the mean temperature (TG variable in E-OBS) in all days of the year, or in the days pertaining to each season (December, January and February were allocated to winter; March, April and May to spring; June, July and August to summer; and September, October and November to fall). These grid cells values are aggregated to the SHARE region through both the median and the mean.

Heating and Cooling Degree days

Following the EUROSTAT definitions (https://ec.europa.eu/eurostat/cache/metadata/en/nrg_chdd_esms.htm), at each grid cell we calculate the number of heating degree days (HDD) and cooling degree days (CDD) using the average temperature from the E-OBS dataset (TG variable). Thus, for HDD, we sum over a year, for each grid cell, the differences between 18ºC and the recorded mean daily temperatures, for every day when the temperature in that grid cell was equal or below 15ºC (average temperature coming from TG variable of E-OBS). For CDD, the process is analogous, except we sum the differences between the recorded mean daily temperature and 21ºC, only for those days where the mean temperature was above 24ºC.

Each grid cell thus has, for each year, an HDD and a CDD index. These are aggregated to the SHARE regions through both the median and the mean, as with the remaining variables.

Radiation

The 0.1° gridded E-OBS dataset provides data on daily radiation starting in 1950 through variable QQ. For each grid cell, we calculate for any given year, the average of the radiation over all the days in that year, or in the days pertaining to each season. These grid cell values are aggregated to the SHARE region through both the median and the mean.

Precipitation

For precipitation we likewise provide yearly variables and cumulative variables calculated from them, starting from the E-OBS dataset, resorting to daily near-surface precipitation (E-OBS variable RR). At each grid cell, we calculate the number of days in each year where the sum of precipitation exceeds 10 mm and 20 mm - heavy and very heavy precipitation days-, as defined in the Agroclimatic indicators datasets part of the C3S Global Agriculture Sectoral Information Systems (SIS). As with temperature variables, these are georeferenced to SHARE regions, and aggregated using the median and mean, alongside the standard deviation to analyze intra-region variation.

Pollution data

The variables considered for pollution relate to the four most explored pollutants in the context of health: particulate matter 2.5 microns (in diameter) ($PM_{2.5}$), particulate matter 10 microns (PM_{10}), ozone (O_3) and nitrogen dioxide (NO_2) (as put forward in the WHO Review of evidence on health aspects of air pollution[S1]).

Concentration

For $PM_{2.5}$, PM_{10} and NO_2, there is limited evidence for the existence of a threshold below which health effects are negligible. Negative health outcomes have been found at very low concentrations (WHO 2014). We therefore resort to yearly average exposures, starting from the dataset CAMS global reanalysis (EAC4) on monthly averaged fields whose first year is 2003.

The original CAMS EAC4 monthly dataset resolution is 0.75° X 0.75°. We disaggregate the dataset into 0.1° X 0.1° through bilinear interpolation, and, at the grid cell level, take the average of the 12 months of each year. As done with the temperature dataset, each grid cell is associated with the SHARE region when its centroid falls within the region boundary, and the three variables, mean, median and standard deviation, are then constructed.

For O_3, the literature documents mixed evidence on the existence of thresholds. Several papers find an association between health outcomes and summer ozone concentration, but not winter season concentration; a finding attributed to the existence of a threshold by some studies or due to confounding effects or seasonal behavioral differences[S2]. Other studies that specifically analyze the threshold question arrive to different conclusions (e.g., evidence of thresholds is found in some studies[S3] but not in others[S4]). We follow the recent literature on long-term effects of ozone exposure and operate with yearly averages of daily maxima and warm-season averages of daily maxima[S5, S6, S7]. The dataset used is CAMS EAC4[12], from which we use the average O_3 concentration at 3-hour intervals of each day at the surface level, whose first year is 2004. For each day, we keep the maximum of the 6 observations reported, at the grid cell level (after disaggregating the spatial resolution from the gridded 0.75° to 0.1° as mentioned above). We then take either the yearly average or the warm months average (April to September) of the daily maxima, for each grid cell. The grid cells are overlapped with the SHARE regions, as with the temperature datasets, and we calculate the mean, median, and standard deviation at the SHARE region level.

Emissions

The datasets on pollution concentration mentioned begin in 2003 (or in 2004 for O_3), thus, enabling coverage for the regular SHARE waves (which start in 2004), but not for the cumulative exposure. To allow us to go further back in time we use a dataset not on pollution concentration, but on pollutant emissions, the EDGAR v5.0 Global Air Pollutant Emissions[i] dataset, which covers the period 1970-2015[13]. The relevant variable for direct health effects is concentration, thus, the health impacts of emissions will be different across regions, depending, namely, on meteorological conditions and topography. Even so, especially given that emissions are the variables which can be affected policy-wise, considering their (indirect) effects on other variables can be of interest. The variables obtained from EDGAR are estimates of yearly emissions of $PM_{2.5}$ and PM_{10} at the grid cell level which we overlap with SHARE regions to obtain the yearly mean, median and standard deviation at the region level. Information on concentration could also be derived from the EDGAR dataset if combined with advanced chemical transport models (CTMs). The original dataset is available a 0.1° X 0.1° resolution.

Flood events data

For floods, we resort to the DFO dataset[11], which provides information on flood events from 1985 until the present. We report 6 variables: the number of flood events, the number of casualties, the number of displaced individuals, a weighted number of flood events (weighted by an indicator 1, 1.5 or 2, representing the severity of the flood event), the total days during which there were floods events, and the weighted total days (weighted by an indicator 1, 1.5 or 2 representing the severity of the flood event).

The variables correspond to whether the individual was living in a region considered in the dataset to be affected by the flood event (more specifically, if the region where the individual was living overlaps with the region provided as 'affected' in the DFO dataset). Since depending on the country, individuals might report a NUTS2 or NUTS1 region, other 12 variables are created. The first 6 refer to whether the NUTS1 region where the individual resided was affected by flood events and the latter 6 to whether the NUTS2 region where the individual resided was affected by flood events.

Regional aggregation and population weighting

We identify households' location through the SHARE regions reported in the retrospective accommodation waves 3 and 7, or through the NUTS in which the household was located at the moment of sampling in the regular waves. The latter is reported in the housing modules of the regular panel waves. We use information from the housing modules on whether individuals changed house to expand forward regional information.

SHARE regions are mostly NUTS2 (Austria, Bulgaria, Croatia, Czechia, Denmark, Finland, Greece, Hungary except for Budapest and Pest, which are reported together as the NUTS1 region of Central Hungary, Italy, Latvia, Lithuania, Poland, Portugal, Romania, Slovakia, Slovenia, Spain and Sweden) with a few countries reporting NUTS1 only (Belgium, France, Germany and one region of Hungary, Central Hungary).[ii]

---

[i] https://edgar.jrc.ec.europa.eu/gallery?release=v50_AP&substance=PM10&sector=TOTALS

[ii] Wave 3 was conducted in almost all countries in 2008/2009 while Wave 7 was conducted in 2017. This would, at first, lead us to use NUTS2006 and NUTS2016 respectively. In practice, the SHARE regions indicated by respondents are consistent from Wave 3 to Wave 7, i.e., they do not change even if there were changes in the NUTS structure. France and Poland are the two examples – there are region changes in the NUTS, but not

Whenever individuals lived in a country different to that in which they were now sampled, we do not know in which region they lived, but only the country. Country-level information is considered too aggregate to provide useful environmental exposure measures. Thus, for periods where respondents were outside the country, we do not have any environmental information. Cumulative exposure variables, therefore, do not consider such years. Averages which explicitly consider this fact can be calculated by dividing cumulative exposures by the number of years for which there is information (which excludes the years when individuals were abroad). We provide the variables necessary for users to build said averages.

From gridded raw datasets, we generate transformed variables at the grid cell level, as explained in the previous sections. We finally aggregate them to the SHARE regions: we detect in which SHARE region the grid cells are located by overlaying them with a shapefile of the SHARE regions, constructed resorting to EUROSTAT NUTS shapefiles (downloadable from EUROSTAT) and to a shapefile of Luxembourg cantons (downloadable from data.public.lu, see SI for more details on the NUTS classifications used.) For climate and pollution variables we provide unweighted variables and population-weighted variables. For population-weighted variables, we resort to the historical gridded population dataset from ISIMIP[iii], which provides annual population estimates for 1901-2020. Weighting is done at the moment of regional aggregation.

A second version of the dataset, currently undergoing further robustness checks, explores more granular geographical data. Resorting to the Degree of Urbanization DEGURBA methodology (the EU/OECD standard for urbanization classification), we classify each grid cell within a SHARE region as being either part of a city, of towns and suburbs, or of a rural area. We compute for each SHARE region-DEGURBA region pair population-weighted exposure variables. With estimated country-specific weights, we transform these into averages for the five regions indicated by SHARE respondents - big cities, suburbs, large towns, small towns and rural areas.

Cumulative variables

The SHARE dataset is a panel dataset. Environmental hazards might have a cumulative impact on health. Situations which took place at a young age might also only later transpire into health consequences.

We therefore construct cumulative variables of exposure to environmental hazards, reflecting not the exposure to for instance extreme temperatures in the year of a wave, but instead exposure since an individual was born until the wave in question, amongst other cumulative indicators.

If a variable has no prefix, it refers to the exposure to the environmental hazard in the year of the wave. Prefixes starting with ‘s’ correspond to a rolling sum of exposure, with the simple ‘s_’ corresponding to the rolling sum of exposure from birth (or from the oldest year available) up until the year of the wave in question.

---

in the SHARE regions, which remain with a direct correspondence of names to NUTS2006. Therefore, for the two countries, we resort to NUTS2006 (shapefile NUTS2013 since there was no change to the NUTS boundaries of the two countries from NUTS2006 to NUTS2013). For the remaining countries, we resort to the NUTS2016 shapefile.

[iii] https://data.isimip.org/datasets/fc1e4a06-bd4a-4044-b8e6-46ce86346489/

The prefixes starting with ‘y’ are simple sums instead of rolling sums; they correspond to total exposure during certain, relevant, years. For early age exposure, ‘y5_’, ‘y10_’ and ‘y15_’ correspond to total exposure during the first 5, 10 and 15 years of age. ‘yjob_’ corresponds to exposure during the years at current job or at the most recent job. We also generate variables for exposure to environment in the years preceding periods of ill health during adulthood. Respondents indicate up to 3 periods where they experienced ill health, specifying the start and end (more details in Appendix 2). For individuals indicating illness periods, we construct variables with prefix ‘yill1_’, ‘yill2_’ and ‘yill3_’ denoting exposure during the years of illness periods 1,2 and 3 respectively. We construct variables with prefix ‘y1bf_’, ‘y3bf_’ and ‘y5bf_’ to represent exposure to hazards during the 1 year, the 3 years and the 5 years preceding the start of each illness period.

We generate cumulative variables since birth for 6 of the 16 temperature bins, on the low extremes and on the high extremes, i.e., for temperatures below 5ºC, between -5ºC and -2.5 ºC and between -2.5 ºC and 0 ºC; and for temperatures between 25 ºC and 27.5 ºC, between 27.5 ºC and 30 ºC, and above 30 ºC. Other bins can be made available on request. On the temperature variables, we report cumulative exposure since birth for CDD and HDD. Cumulative variables since birth are also available for precipitation. We report cumulative variables for flood variables as well.

As auxiliary variables, we report the rolling sum of the number of years for which cumulative measures were computed. We choose to provide both cumulative exposures and years for which cumulative exposure is available, instead of only averages, since even for the same variable, the information for the same number of years for all individuals is not available. This is for two reasons: i) individuals who were born before the years where the environmental variables start and ii) periods in which individuals were outside their country of interview. By providing both cumulative and years available, averages can be readily computed through their ratio, if averages are the variables of interest, and simultaneously, subsets of the sample based on the number of years available (e.g., necessarily all years since birth) can be analyzed separately.

We report as well average spring, summer, fall, winter, and yearly temperatures and average radiation, since birth and during the first 5, 10 and 15 years of life. For these, we directly provide these averages alongside the rolling sum of the number of years, instead of cumulative exposure as we do for the remaining (count) variables.

Table S1 Environmental variables list

Variable name	Variable description
**Temperature Variables**	
Bins	
[tn/tg/tx]_neg5_[median/mean/w][none/_t1bf/_t2bf] (3*2*3 vars)	No. days [min/avg/max] temp. below -5ºC<br>([median/mean/population weighted mean] grid cells) at [year of wave/year before/2years before]
[tn/tg/tx]_neg5_neg2p5_[median/mean/w][none/_t1bf/_t2bf] (3*2*3 vars)	No. days [min/avg/max] temp. bt -5 and -2.5ºC ([median/mean/population weighted mean] grid cells) at [year wave/year before/2years before]

[tn/tg/tx]_neg2p5_0_[median/mean/w][none/_t1bf/_t2bf] (3*2*3 vars)	No. days [min/avg/max] temp. bt -2.5 and 0ºC ([median/mean/population weighted mean] grid cells) at [year wave/year before/2years before]
[tn/tg/tx]_0_2p5_[median/mean/w][none/_t1bf/_t2bf] (3*2*3 vars)	No. days [min/avg/max] temp. bt 0 and 2.5ºC ([median/mean/population weighted mean] grid cells) at [year wave/year before/2years before]
[tn/tg/tx]_2p5_5_[median/mean/w][none/_t1bf/_t2bf] (3*2*3 vars)	No. days [min/avg/max] temp. bt 2.5 and 5ºC ([median/mean/population weighted mean] grid cells) at [year wave/year before/2years before]
[tn/tg/tx]_5_7p5_[median/mean/w][none/_t1bf/_t2bf] (3*2*3 vars)	No. days [min/avg/max] temp. bt 5 and 7.5ºC ([median/mean/population weighted mean] grid cells) at [year wave/year before/2years before]
[tn/tg/tx]_7p5_10_[median/mean/w][none/_t1bf/_t2bf] (3*2*3 vars)	No. days [min/avg/max] temp. bt 7.5 and 10ºC ([median/mean/population weighted mean] grid cells) at [year wave/year before/2years before]
[tn/tg/tx]_10_12p5_[median/mean/w][none/_t1bf/_t2bf] (3*2*3 vars)	No. days [min/avg/max] temp. bt 10 and 12.5ºC ([median/mean/population weighted mean] grid cells) at [year wave/year before/2years before]
[tn/tg/tx]_12p5_15_[median/mean/w][none/_t1bf/_t2bf] (3*2*3 vars)	No. days [min/avg/max] temp. bt 12.5 and 15ºC ([median/mean/population weighted mean] grid cells) at [year wave/year before/2years before]
[tn/tg/tx]_15_17p5_[median/mean/w][none/_t1bf/_t2bf] (3*2*3 vars)	No. days [min/avg/max] temp. bt 15 and 17.5ºC ([median/mean/population weighted mean] grid cells) at [year wave/year before/2years before]
[tn/tg/tx]_17p5_20_[median/mean/w][none/_t1bf/_t2bf] (3*2*3 vars)	No. days [min/avg/max] temp. bt 17.5 and 20ºC ([median/mean/population weighted mean] grid cells) at [year wave/year before/2years before]
[tn/tg/tx]_20_22p5_[median/mean/w][none/_t1bf/_t2bf] (3*2*3 vars)	No. days [min/avg/max] temp. bt 20 and 22.5ºC ([median/mean/population weighted mean] grid cells) at [year wave/year before/2years before]
[tn/tg/tx]_22p5_25_[median/mean/w][none/_t1bf/_t2bf] (3*2*3 vars)	No. days [min/avg/max] temp. bt 22.5 and 25ºC ([median/mean/population weighted mean] grid cells) at [year wave/year before/2years before]
[tn/tg/tx]_25_27p5_[median/mean/w][none/_t1bf/_t2bf] (3*2*3 vars)	No. days [min/avg/max] temp. bt 25 and 27.5ºC ([median/mean/population weighted mean] grid cells) at [year wave/year before/2years before]

[tn/tg/tx]_27p5_30_[median/mean/w][none/_t1bf/_t2bf] (3*2*3 vars)	No. days [min/avg/max] temp. bt 27.5 and 30ºC ([median/mean/population weighted mean] grid cells) at [year wave/year before/2years before]
[tn/tg/tx]_g30_[median/mean/w][none/_t1bf/_t2bf] (3*2*3 vars)	No. days [min/avg/max] temp. above 30ºC ([median/mean/population weighted mean] grid cells) at [year wave/year before/2years before]
Average temperatures	
temperature_[median/mean/w][none/_t1bf/_t2bf] (2*3 vars)	Avg. daily mean temperature ([median/mean/population weighted mean] grid cells) at [year wave/year before/2years before]
summer_[median/mean/w][none/_t1bf/_t2bf] (2*3 vars)	Avg. summer daily mean temperature ([median/mean/population weighted mean]] grid cells) at [year wave/year before/2years before]
spring_[median/mean/w][none/_t1bf/_t2bf] (2*3 vars)	Avg. spring daily mean temperature ([median/mean/population weighted mean] grid cells) at [year wave/year before/2years before]
fall_[median/mean/w][none/_t1bf/_t2bf] (2*3 vars)	Avg. fall daily mean temperature ([median/mean/population weighted mean] grid cells) at [year wave/year before/2years before]
winter_[median/mean/w][none/_t1bf/_t2bf] (2*3 vars)	Avg. winter daily mean temperature ([median/mean/population weighted mean] grid cells) at [year wave/year before/2years before]
CDD/HDD	
CDD_[median/mean/w][none/_t1bf/_t2bf] (2*3 vars)	EUROSTAT Cooling degree days index ([median/mean/population weighted mean] grid cells) at [year wave/year before/2years before]
HDD_[median/mean/w][none/_t1bf/_t2bf] (2*3 vars)	EUROSTAT Heating degree days index ([median/mean/population weighted mean] grid cells) at [year wave/year before/2years before]
**Radiation Variables**	
radiation_[median/mean/w][none/_t1bf/_t2bf] (2*3 vars)	Average daily radiation ([median/mean/population weighted mean] grid cells) at [year wave/year before/2years before]
radiation_spring_[median/mean/w][none/_t1bf/_t2bf] (2*3 vars)	Average daily radiation in spring months of year ([median/mean/population weighted mean] grid cells) at [year wave/year before/2years before]

radiation_summer_[median/mean/w][none/_t1bf/_t2bf] (2*3 vars)	Average daily radiation in summer months of year ([median/mean/population weighted mean] grid cells) at [year wave/year before/2years before]
radiation_fall_[median/mean/w][none/_t1bf/_t2bf] (2*3 vars)	Average daily radiation in fall months of year ([median/mean/population weighted mean] grid cells) at [year wave/year before/2years before]
radiation_winter_[median/mean/w]_[t0/t_1bf/t_2bf] (2*3 vars)	Average daily radiation in winter months of year ([median/mean/population weighted mean] grid cells) at [year wave/year before/2years before]
**Precipitation Variables**	
prec10_[median/mean/w][none/_t1bf/_t2bf] (2*3 vars)	No. days total precipitation above 10mm ([median/mean/population weighted mean] grid cells) at [year wave/year before/2years before]
prec20_[median/mean/w][none/_t1bf/_t2bf] (2*3 vars)	No. days total precipitation above 20mm ([median/mean/population weighted mean] grid cells) at [year wave/year before/2years before]
**Flood Variables**	
fl_no_floods_[SHARE/NUTS1/NUTS2] [none/_t1bf/_t2bf] (3*3 vars)	No. flood events in SHARE region / NUTS1 region / NUTS2 region at [year wave/year before/2years before]
fl_tot_dead_[SHARE/NUTS1/NUTS2] [none/_t1bf/_t2bf] (3*3 vars)	No. casualties of flood events in SHARE region / NUTS1 region / NUTS2 region at [year wave/year before/2years before]
fl_tot_displaced_[SHARE/NUTS1/NUTS2] [none/_t1bf/_t2bf] (3*3 vars)	No. displaced by flood events in SHARE region / NUTS1 region / NUTS2 region at [year wave/year before/2years before]
fl_weighted_floods_[SHARE/NUTS1/NUTS2] [none/_t1bf/_t2bf] (3*3 vars)	Weighted No. of flood events in SHARE region / NUTS1 region / NUTS2 region at [year wave/year before/2years before]
fl_tot_days_[SHARE/NUTS1/NUTS2] [none/_t1bf/_t2bf] (3*3 vars)	No. days of flood events in SHARE region / NUTS1 region / NUTS2 region at [year wave/year before/2years before]
fl_weighted_[days_SHARE/NUTS1/NUTS2] [none/_t1bf/_t2bf] (3*3 vars)	Weighted No. days of flood events in SHARE region / NUTS1 region / NUTS2 region at [year wave/year before/2years before]
**Pollution vars**	

Concentration	
conc_pm2p5_[median/mean/w][none/_t1bf/_t2bf] (2*3 vars)	Avg monthly concentration $PM_{2.5}$ ([median/mean/population weighted mean] grid cells) at [year wave/year before/2years before]
conc_pm10_[median/mean/w][none/_t1bf/_t2bf] (2*3 vars)	Avg monthly concentration PM_{10} ([median/mean/population weighted mean] grid cells) at [year wave/year before/2years before]
conc_no2_[median/mean/w][none/_t1bf/_t2bf] (2*3 vars)	Avg monthly concentration NO_2 ([median/mean/population weighted mean] grid cells) at [year wave/year before/2years before]
conc_yearly_o3_[median/mean/w][none/_t1bf/_t2bf] (2*3 vars)	Avg daily max O_3 concentration ([median/mean/population weighted mean] grid cells) at [year wave/year before/2years before]
Conc_warm_o3_[median/mean/w][none/_t1bf/_t2bf] (2*3 vars)	Avg daily max O_3 concentration in warm months (Apr-Sep) ([median/mean/population weighted mean] grid cells) at [year wave/year before/2years before]
Emissions	
emissions_PM25_[median/mean/w][none/_t1bf/_t2bf] (2*3 vars)	Yearly emissions of $PM_{2.5}$ ([median/mean/population weighted mean] grid cells) at [year wave/year before/2years before]
emissions_PM10_[median/mean/w][none/_t1bf/_t2bf] (2*3 vars)	Yearly emissions of PM_{10} ([median/mean/population weighted mean] grid cells) at [year wave/year before/2years before]

The cumulative variables are created using the yearly variables; therefore, their names are the same, but with added prefixes which indicate over what period are the cumulative measures taken.

Table S2 Cumulative environmental variables list

**Main exposure variables**			
**Cumulative variable prefix**	**Prefix meaning**	**Yearly Variables for which the cumulative measure is calculated**	**Module**
s_	Rolling sum since birth (or earliest available year) until present wave	Bin variables (1920), HDD (1920), CDD (1920), Precipitation Variables	life_module

		(1920), Flood Variables (1985)	
avg_	Rolling average since birth (or earliest available year) until present wave	Average temperature variables (1920), Radiation Variables (1950), Concentration Variables (2003/2004), Emissions Variables (1970),	life_module
y5_ / y10_ / y_15	Cumulative exposure during the first 5/10/15 years of life	Bin variables, HDD, CDD, Precipitation Variables (no flood variables since no individual is born after 1970, thus, not 15 after 1985)	young_age_ module
avg5_ / avg10_ / avg15_	Average during the first 5/10/15 years of life	Average temperatures variables, Radiation Variables, (no concentration variables), Emission Variables	young_age_ module
yjob_	Cumulative exposure during the most recent job	Bin variables, HDD, CDD, Precipitation Variables, (no flood variables)	job_module
avgjob_	Average exposure during the most recent job	Average temperature variables (1920), Radiation Variables (1950), Concentration Variables (2003/2004), Emissions Variables (1970),	job_module
yill[1/2/3]_	Cumulative exposure during illness period 1/2/3	Bin variables, HDD, CDD, Precipitation Variables, Flood variables	illness_durin g_module
avgill[1/2/3]_	Average exposure during illness period [1/2/3]	Average temperature variables (1920), Radiation Variables (1950), Concentration Variables (2003/2004), Emissions Variables (1970),	illness_durin g_module

y[1/3/5]bf_[1/2/3]_	Cumulative exposure during the [1/3/5] year(s) preceding illness period [1/2/3]	Bin variables, HDD, CDD, Precipitation Variables, Flood variables	illness_before_module
avg[1/3/5]bf_[1/2/3]_	Average exposure during the [1/3/5] year(s) preceding illness period [1/2/3]	Average temperature variables (1920), Radiation Variables (1950), Concentration Variables (2003/2004), Emissions Variables (1970),	illness _before_module
**Auxiliary variables (denominator for averages)**			
rol_years_exposure_ [temp / prec/rad/ fl[SHARE/NUTS1/NUTS2] /conc_noto3/ conc_o3/emissions]	Rolling sum of non-empty years of [temperature/ precipitation/ radiation / flood pertaining to [SHARE region /NUTS1 region/NUTS2 region] / non-O_3 concentration/ O_3 concentration/emissions] variables	All variables	life_module
tot_years_exposure_ [temp/prec/ rad/ fl[SHARE/NUTS1/NUTS2]/ conc_noto3 /conc_o3/emissions]	Maximum of rol_years_exposure_ [temp/prec/ rad/fl[SHARE/NUTS1/NUTS2] /conc_noto3 /conc_o3/emissions]	All variables	life_module
years_present_ [temp/prec/rad/emissions] _ outof[5/10/15]	Years for which there is information on [temperature /precipitation /radiation/emissions] variables out of the first [5/10/15] years of life	All variables except flood and concentration variables (do not go back in time sufficiently to catch the first 15 years of life of respondents)	young_age_module

job_years_exposure_ [temp/prec/rad/emissions /con_noto3/conc_o3]	Years in which individual was at most recent job for which there is information on [temperature /precipitation /radiation/emissions /non-ozone concentration/ozone concentration] variables	All variables except flood variables (flood events during years at most recent job not considered a variable of interest)	job_module
Ill_years_exp_dur[1/2/3]_ [temp/prec /rad/emissions/ fl[SHARE/NUTS1/NUTS2]/ conc_noto3/conc_o3/emis sions]_	Years for which during period of illness [1/2/3] there is info on [temperature/precipitatio n/radiation/ emissions/floods at [SHARE level/NUTS1 level/NUTS2 level]/non-ozone concentration/ozone concentration/emissions] variables	All variables	illness_durin g_module
ill_y_exp_[1/3/5]_bf_[1/2/ 3]_ [temp/prec /rad/emissions/ fl[SHARE/NUTS1/NUTS2]/ con_noto3/con_o3/emissio ns]	Years for which there is information out of the [1/3/5] years before illness period [1/2/3] on [temperature/precipitatio n/ radiation/ Emissions/floods at [SHARE level/NUTS1 level/NUTS2 level]/not-ozone concentration/ozone concentration/emissions] variables	All variables	illness_befor e_module

Appendix 2. Details on illness variables

Table S3 Generated morbidity variables in the SHARE-ENV dataset, modules illness_before and illness_during

Variable	Variable Description
ill_length_[1/2/3]	Length of illness period 1/2/3
ill_age_onset_[1/2/3	Age of onset of illness period 1/2/3
ill_start_[1/2/3]	Year when illness period 1/2/3 started
ill_end_[1/2/3]	Year when illness period 1/2/3 ended
Ill_any_issue[1/2/3]	Any issue in period 1/2/3
Ill_any_env_related_issue[1/2/3]	Any environment-related issue in period 1/2/3
[Environment-related illness name][1/2/3]	Whether it was [angina or heart attack/stroke/asthma/other respiratory problems/migraines/emotional distress/fatigue/infectious diseases/allergies] (one of) the issue(s) responsible for illness period 1/2/3

Note: Environment-related issues are angina or heart attack, stroke, asthma, (other) respiratory problems, migraines, emotional distress, fatigue, infectious diseases and allergies. These variables are provided as part of the 'illness_before_module' and 'illness_during_module'. Respondents report on only up to three periods of illness, coded as 1/2/3 respectively.

Appendix 3. Extended regression results

Table S4 Extensive regression results of Main Text Table 1

Variables	1. Ever experienced breathlessness		2. Young age (<15) perceived reported health (1=poor; 5= excellent)		3. Uncomfortable job		4. High cognitive decline (1=yes, 0=no)	
**Exposure Variables**	**Avg. $PM_{2.5}$ conc. median (μg/m³)**	0.00190***<br>(0.000691)	**Avg. first 15 years exposure to negative temp. (# days)**	0.000182<br>(0.000384)	**Average winter temp.**	0.00748***<br>(0.00131)	**Difference avg. $PM_{2.5}$ (μg/m³)**	0.00374***<br>(0.000747)
	**Avg. Cum. lifetime exposure to negative temp. (# days)**	4.42E-06<br>(0.000091)	**Avg. first 15 years exposure to temp. > 30°C (# days)**	0.00283*<br>(0.00151)	**Average summer temp.**	-0.00268***<br>(0.000989)	**Difference in heating degree days**	-7.11e-06 ***<br>(5.31e-07)
	**Avg. Lifetime exposure to temp.> 30°C (# days)**	-0.000436*<br>(0.000244)	**Avg. first 15 years solar radiation (W/m²)**	0.00219*<br>(0.00134)	Average radiation	0.000148<br>(0.000370)		
**Exposure x Individual characteristics**					**Job is physical x average winter temp.**	-0.0118***<br>(0.00156)		
					**Job is physical x average summer temp.**	0.00831***<br>(0.00136)		
					**Job is physical x average radiation**	0.00135***<br>(0.000264)		
**Whether job is physical**								
Job is physical						-0.00143<br>(0.0302)		
**Occupation and educ. fixed effects**								
Occupation ISCO codes (1digit)						Y		

Parental ISCO codes (1digit)		N/A	Y			
Mother ISCED educ. level			0.0154			
			(0.00940)			
Father ISCED educ. level			0.0171**			
			(0.00827)			
ISCED educ. level 2					-0.0145	-0.0346***
					(0.00895)	(0.00623)
ISCED educ. level 3					-0.0435***	-0.0633***
					(0.00839)	(0.00573)
ISCED educ. level 4					-0.0622***	-0.0620***
					(0.0124)	(0.0112)
ISCED educ. level 5					-0.0802***	-0.0938***
					(0.00922)	(0.00620)
ISCED educ. level 6					-0.121***	-0.123***
					(0.0207)	(0.0150)
**Socioeconomic conditions controls**						
Household Net worth						
Household Income (current/average)	average	-5.09e-07***		average	-3.72e-07***	
		(0.0000000807)			(1.11E-07)	
House at 15 years had no basic amenities			-0.119***			
			(0.0277)			
Rooms / people when 10 years old			0.0333*			
			(0.0201)			
Ever poor in childhood			-0.165***			
			(0.0210)			
% of time in urban area			-0.00915			
			(0.0217)			
Physical harm in childhood			-0.102***			
			(0.0163)			
Loneliness in childhood			-0.281***			
			(0.0205)			
Type of area dummies		N	N		N	Y
Ever moved dummy						-0.0129***
						(0.00393)
**Behavioral variables**						

BMI (Body Mass Index)	average	0.00974***			
		(0.000482)			
Ever smoked		0.0391***			
		(0.00387)			
Sports more than once a week		0			
		0			
Sports once a week		7.12E-05			
		(0.0057)			
Sports once to three times a month		0.0097			
		(0.0062)			
Sports hardly ever		0.0802***			
		(0.00484)			
**Health variables**					
Lagged health status		N/A	N/A	N/A	-0.000424
					(0.00598)
					-0.00854
					(0.00577)
					-0.0212***
					(0.00657)
					-0.0512***
					(0.00934)
Lagged depression score		N/A	N/A	N/A	0.00953***
					(0.00107)
Diff. in depression score		N/A	N/A	N/A	0.0107***
					(0.00102)
Lagged cognitive score		N/A	N/A	N/A	0.0177***
					(0.000601)
Born with an illness		0.129***	-0.661***		
		(0.0276)	(0.0906)		
**Job comfort level**					
Job is uncomfortable [strongly disagree]		0			
		0			
Job is uncomfortable [disagree]		0.0188***			
		(0.00465)			
Job is uncomfortable [agree]		0.0392***			
		(0.00565)			
Job is uncomfortable [strongly agree]		0.0623***			
		(0.00718)			
**Fixed at birth controls**					

Female	N	N	N	-0.0314***
				(0.00359)
Current Age	0.00478***		lagged	0.00633***
	(0.000219)			(0.000209)
Year of Birth		0.0105***		
		(0.00113)		
**Country Fixed Effects**	**Y**	**Y**	**Y**	**Y**
**Year Fixed Effects**	**N/A**	**N/A**	**N/A**	**N**
Observations	33,482	16,040	43,717	40,050
R-squared	0.080	0.081	0.205	0.064

Robust standard errors in parentheses.

*** p<0.01, ** p<0.05, * p<0.1

Table S5 Effect of temperature exposure on old age health at first wave of participation (younger age) and at last wave of participation (older age)

	**Old age (>49) perceived reported health (1=poor; 5= excellent)**	
	First wave of participation	Last wave of participation
**Avg. Lifetime exposure to temp. > 27.5°C (# days)**	-0.000467	-0.00182***
	(0.000490)	(0.000498)
**Avg. Lifetime exposure to negative temp. (# days)**	-0.000824***	-0.000153
	(0.000259)	(0.000244)
**Occupation and educational effects**		
ISCED educ. level 2	0.0809***	0.0582***
	(0.0160)	(0.0154)
ISCED educ. level 3	0.207***	0.138***
	(0.0149)	(0.0141)
ISCED educ. level 4	0.327***	0.169***
	(0.0296)	(0.0255)
ISCED educ. level 5	0.399***	0.249***
	(0.0164)	(0.0159)
ISCED educ. level 6	0.391***	0.290***
	(0.0515)	(0.0468)
Household Net worth	7.94e-08***	1.25e-07***
	(1.90e-08)	(2.41e-08)
Household Income (current/average)	8.84e-07***	2.15e-06***
	(1.70e-07)	(2.70e-07)

**Behavioral variables**		
BMI (Body Mass Index)	-0.0304***	-0.0285***
	(0.00109)	(0.00105)
Ever smoked	-0.0788***	-0.0651***
	(0.00942)	(0.00897)
Sports more than once a week	0	0
	(0)	(0)
Sports once a week	-0.137***	-0.0667***
	(0.0135)	(0.0157)
Sports one to three times a month	-0.173***	-0.0884***
	(0.0155)	(0.0168)
Sports hardly ever	-0.485***	-0.378***
	(0.0113)	(0.0126)
Depression	-0.418***	-0.448***
	(0.00976)	(0.0118)
Job is uncomfortable [strongly disagree]	0	0
	(0)	(0)
Job is uncomfortable [disagree]	-0.104***	-0.0903***
	(0.0116)	(0.0110)
Job is uncomfortable [agree]	-0.189***	-0.171***
	(0.0136)	(0.0130)
Job is uncomfortable [strongly agree]	-0.231***	-0.228***
	(0.0170)	(0.0162)
Female	-0.00493	0.0561***
	(0.00975)	(0.00939)
Year of Birth	0.0152***	0.0206***
	(0.000549)	(0.000532)
**Country Fixed Effects**	**Y**	**Y**
**Year Fixed Effects**	**Y**	**Y**
Observations	41,133	45,123
R-squared	0.272	0.223

Robust standard errors in parentheses.

*** p<0.01, ** p<0.05, * p<0.1

Table S6 Effect of $PM_{2.5}$ concentration on cognitive decline, fixed effects regression

	Difference in Cognitive score
**avg. $PM_{2.5}$ conc. median ($\mu g/m^3$)**	-0.00668***
	(0.00114)
Heating degree days	-2.02e-07
	(3.90e-07)
Age	0.00102
	(0.000764)
Depression score	-0.00767***
	(0.00111)
Household income	2.71e-08
	(2.73e-08)
Sports more than once a week	0
	(0)
Sports once a week	-0.00295
	(0.00494)
Sports one to three times a month	0.00249
	(0.00595)
Sports hardly ever	-0.0108**
	(0.00442)
**Individual fixed effects**	**Y**
Observations	98,861
R-squared	0.005

Robust standard errors in parentheses.

*** p<0.01, ** p<0.05, * p<0.1

Appendix 4. Summary statistics

Table S7 Summary statistics of variables in Table S4

VARIABLES	N	mean	s.d.	min	max
**Young age health**					
Young age (<15) perceived reported health (1=poor; 5= excellent)	90,103	3.853	1.035	1	5
Avg. first 15 years solar radiation (W/m^2)	59,602	136.9	24.14	58.52	222.2
Avg. first 15 years exposure to temperature > 30°C (# days)	71,230	10.87	15.79	0	86.07
Avg. first 15 years exposure to negative temperature (# days)	71,230	87.87	45.77	0	237.4
Loneliness in childhood	61,212	0.203	0.402	0	1
Physical harm in childhood	56,245	0.401	0.490	0	1
Ever poor in childhood	59,183	0.309	0.462	0	1
House at 15 years had no basic amenities	89,747	0.321	0.467	0	1
Rooms / people when 10 years old	88,139	0.714	0.441	0	16.67
Born with an illness	90,103	0.0104	0.101	0	1
Father ISCED educ. level	41,006	2.371	1.331	1	6
Mother ISCED educ. level	40,414	1.965	1.096	1	6
% of time in urban area	69,825	0.183	0.373	0	1
Parental ISCO codes (1digit)	90,103	6.748	3.107	1	11
Year of Birth	90,078	1,947	10.54	1910	1995
**Ever experienced breathlessness**					
Ever experienced breathlessness	84,171	0.165	0.371	0	1
Avg. $PM_{2.5}$ conc. median (µg/m^3)	108,460	15.93	4.825	3.059	36.87
Avg. Lifetime exposure to temperature > 30°C (# days)	108,464	14.40	16.93	0	115
Avg. Cum. lifetime exposure to negative temperature (# days)	108,464	79.48	43.83	0	227.1
Household Income (average)	139,010	25,384	41,728	0	5.038e+06
Born with an illness	206,725	0.00993	0.0991	0	1
BMI (Body Mass Index, average)	136,226	27.66	4.885	12.40	99.09
Current Age	124,812	68.82	11.07	22	111
Job is uncomfortable	77,373	2.253	0.987	1	4
Frequency of exercise	119,412	3.023	1.247	1	4
Ever smoked	118,381	0.461	0.498	0	1
**Uncomfortable job**					
Uncomfortable job	57,617	0.393	0.489	0	1
Average radiation	66,337	135.1	24.53	40.37	223.5
**Average summer temperature**	66,451	16.41	3.746	-5.867	33.74
**Average winter temperature**	66,451	4.670	3.716	-12.70	25.77
Physical job	57,657	0.582	0.493	0	1
Household Income (average)	139,010	25,384	41,728	0	5.038e+06
**Cognitive decline**					
High cognitive decline	144,107	0.162	0.368	0	1
Difference avg. $PM_{2.5}$ (µg/m^3)	134,644	3.135	2.389	0.255	23.52
Difference in average summer temperature	133,376	2,608	985.8	21.50	6,489
Lagged cognitive score	216,992	8.913	3.696	0	20
Age (lagged)	206,047	66.77	10.18	15	106
ISCED education level	206,473	2.873	1.418	1	6
Lagged health status	203,727	1.823	1.086	0	4
Lagged depression score	209,283	2.435	2.267	0	12
Diff. in depression score	122,660	0.0180	2.135	-12	12
Gender	523,291	0.529	0.499	0	1
Type of area	216,109	3.441	1.443	1	5
Ever moved region	296,090	0.277	0.448	0	1

Table S8 Summary statistics of variables in Table S5

VARIABLES	N	mean	s.d.	min	max
**Constant across all observations**					
Gender	81,806	1.554	0.497	0	1
Year of birth	81,806	1,946	10.28	1,902	1,980
Job is uncomfortable	55,468	2.177	0.977	1	4
**First observation**					
Old age (>49) perceived reported health (1=poor; 5= excellent)	81,806	2.885	1.092	1	5
Avg. lifetime exposure to negative temperature (# days)	63,929	74.65	42.75	0	228.0
Avg. lifetime exposure to temperature > 27.5°C (# days)	63,929	28.79	26.73	0	147
Ever smoked	80,652	0.455	0.498	0	1
Sports frequency	81,590	2.533	1.335	1	4
BMI (current)	78,946	26.89	4.592	12.76	86.59
Depression score (EUROD)	80,780	0.398	0.490	0	1
Household Income (current)	81,806	34,360	50,546	0	3.712e+06
Household Net worth	81,806	261,255	533,757	-718,856	3.628e+07
**Last observation**					
Old age (>49) perceived reported health (1=poor; 5= excellent)	81,806	2.687	1.069	1	5
Avg. lifetime exposure to negative temperature (# days)	67,454	75.13	42.71	0	227.1
Avg. lifetime exposure to temperature > 27.5°C (# days)	67,454	28.65	26.59	0	147
Ever smoked	81,093	0.454	0.498	0	1
Sports frequency	38,609	2.832	1.311	1	4
BMI (current)	78,683	26.88	4.664	12.46	74.05
Depression score (EUROD)	36,901	0.397	0.489	0	1
Household Income (current)	81,806	15,027	69,562	0	1.004e+07
Household Net worth	81,806	124,386	325,012	-7.240e+06	1.501e+07

Note: Old age subsample with at least two health status observations, 50+ in the first obs.

Table S9 Summary statistics of variables in Table S6

VARIABLES	N	mean	s.d.	min	max
Sports frequency	98,861	2.683	1.323	1	4
Household income	98,861	30,845	67,542	0	1.004e+07
Depression score (EUROD)	98,861	2.371	2.244	0	12
Age	98,861	68.26	9.774	26	103
Diff. in cognitive score (annualized)	98,861	0.0435	0.300	-1	7.500
Cumulative HDD	98,861	169,268	95,738	0	599,069
Avg. $PM_{2.5}$ conc. median (µg/m^3)	98,861	14.38	5.334	0	36.87

Appendix 5. Supplementary references

S1 World Health Organization. *Review of Evidence on Health Aspects of Air Pollution – REVIHAAP Project: Technical Report*; WHO Regional Office for Europe, 2013. https://doi.org/10.1007/BF00379640.

S2 Gryparis, A.; Forsberg, B.; Katsouyanni, K.; Analitis, A.; Touloumi, G.; Schwartz, J.; Samoli, E.; Medina, S.; Anderson, H. R.; Niciu, E. M.; Wichmann, H.-E.; Kriz, B.; Kosnik, M.; Skorkovsky, J.; Vonk, J. M.; Dörtbudak, Z. Acute Effects of Ozone on Mortality from the “Air Pollution and Health. *Am. J. Respir. Crit. Care Med.* **2004**, *170* (10), 1080–1087. https://doi.org/10.1164/rccm.200403-333OC.

S3 Kim, S. Y.; Lee, J. T.; Hong, Y. C.; Ahn, K. J.; Kim, H. Determining the Threshold Effect of Ozone on Daily Mortality: An Analysis of Ozone and Mortality in Seoul, Korea, 1995-1999. *Environ. Res.* **2004**, *94* (2), 113–119. https://doi.org/10.1016/j.envres.2003.09.006.

S4 Bell, M. L.; Peng, R. D.; Dominici, F. The Exposure-Response Curve for Ozone and Risk of Mortality and the Adequacy of Current Ozone Regulations. *Environ. Health Perspect.* **2006**, *114* (4), 532–536. https://doi.org/10.1289/ehp.8816.

S5 Kazemiparkouhi, F.; Eum, K.-D.; Wang, B.; Manjourides, J.; Suh, H. H. Long-Term Ozone Exposures and Cause-Specific Mortality in a US Medicare Cohort. *J. Expo. Sci. Environ. Epidemiol.* **2020**, *30*, 650–658. https://doi.org/10.1038/s41370-019-0135-4.

S6 Lim, C. C.; Hayes, R. B.; Ahn, J.; Shao, Y.; Silverman, D. T.; Jones, R. R.; Garcia, C.; Bell, M. L.; Thurston, G. D. Long-Term Exposure to Ozone and Cause-Specific Mortality Risk in the United States. *Am. J. Respir. Crit. Care Med.* **2019**, *200* (8), 1022–1031. https://doi.org/10.1164/rccm.201806-1161OC.

S7 Malley, C. S.; Henze, D. K.; Kuylenstierna, J. C. I.; Vallack, H. W.; Davila, Y.; Anenberg, S. C.; Turner, M. C.; Ashmore, M. R. Updated Global Estimates of Respiratory Mortality in Adults ≥ 30 Years of Age Attributable to Long-Term Ozone Exposure. *Environ. Health Perspect.* **2017**, *125* (8). https://doi.org/10.1289/EHP1390.
